# Supplementary material for: Metalloproteinases and Hypertrophic Cardiomyopathy: A Systematic Review
Source: Biomolecules. 2023 Apr 11;13(4):665. doi: 10.3390/biom13040665 (PMC10136246; doi:10.3390/biom13040665)
Supplement: Supplementary file 1 [file biomolecules-13-00665-s001.zip › biomolecules-2194366-supplementary.pdf]

Supplemental Material

Metalloproteinases and hypertrophic cardiomyopathy: a systematic review

Supplemental Figures

Figure 1. Preferred Reporting Items for Systematic Reviews and Meta-Analyses (PRISMA) flow diagram………………………………………………………………………..…….pag. 2

Supplemental Tables

Table 1. PICo criteria for inclusion and exclusion of studies……….……….……...…..pag. 4

Table 2. Quality scoring for included papers using Newcastle-Ottawa Scale (NOS)….. pag. 4

Table 3. Characteristics of excluded studies……………………………………………..pag. 5

Figure 1. Preferred Reporting Items for Systematic Reviews and Meta-Analyses (PRISMA) flow diagram.

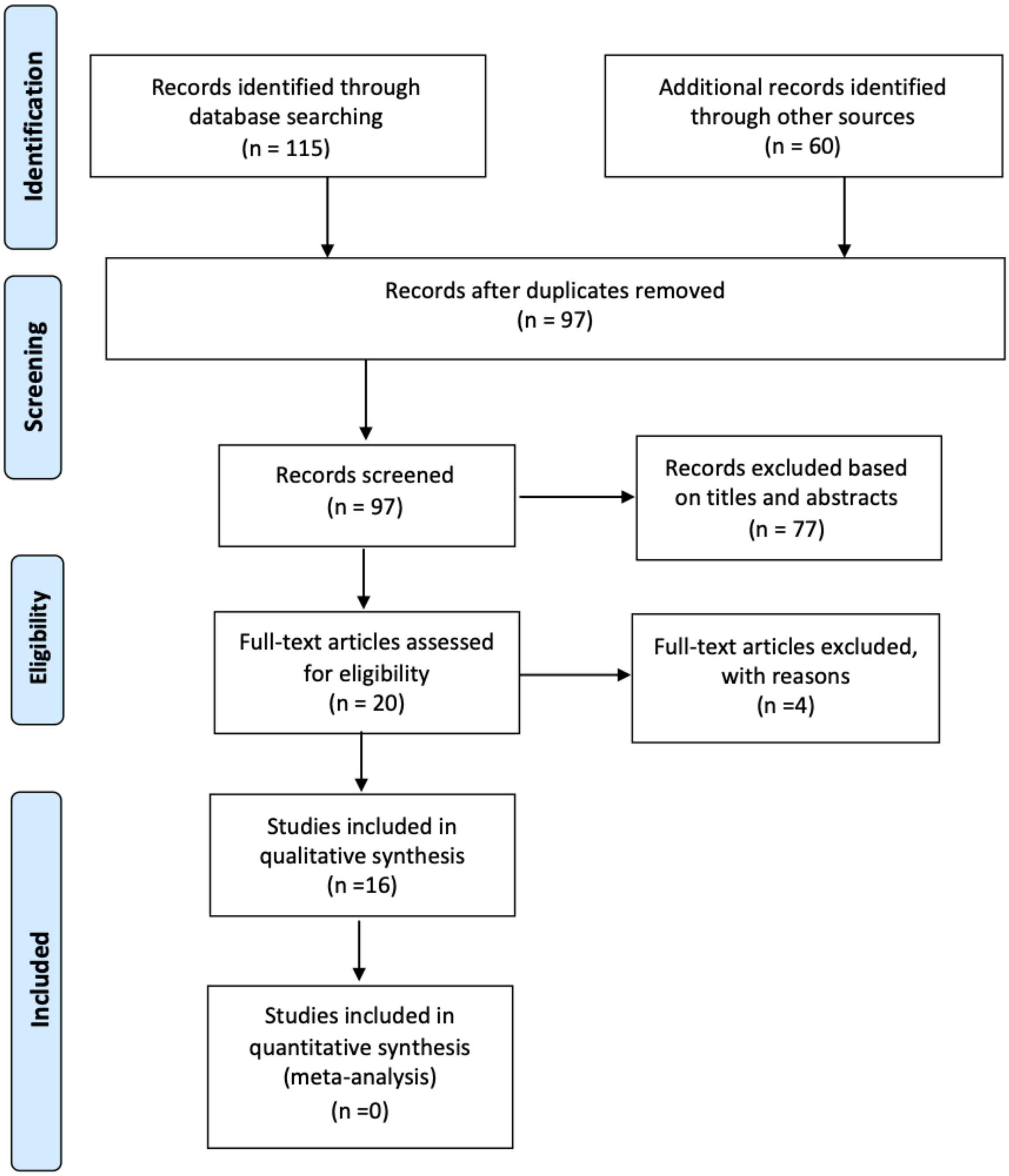

Table 1. PICo criteria for inclusion and exclusion of studies

Parameter	Inclusion criteria	Exclusion criteria
Population/Patient/Problem	Patient with hypertrophic cardiomyopathy	-
Interest	Metalloproteinases analysis	-
Context	Hypertrophic cardiomyopathy	-

Table 2. Quality scoring for included papers using Newcastle-Ottawa Scale

Study (Author, Year)	Selection	Comparability	Outcome	Exposure	*Total*
Bradham, 2002 [10]	3	-	1	–	*4*
Lombardi, 2003 [11]	4	2	1	–	*7*
Fassbach, 2004 [12]	4	2	1	–	*7*
Noji, 2004 [13]	4	2	1	–	*7*
Stroud, 2005 [14]	4	2	1	–	*7*
Roldàn, 2008 [15]	4	2	1	–	*7*
Saura, 2009 [16]	3	-	1	–	*4*
Kitaoka 2010 [17]	3	-	1	–	*4*
Kitaoka, 2011 [18]	3	-	1	–	*4*
Zachariah, 2012 [19]	2	-	1	–	*3*
Fucikova, 2016 [20]	4	2	1	–	*7*
Munch, 2016 [21]	2	-	1	–	*3*
Fernlund, 2017 [22]	4	2	1	–	*7*
Yang, 2019 [23]	3	-	1	–	*4*
Qian, 2020 [24]	3	-	2	–	*5*
Bi, 2021 [25]	3	-	1	–	*4*

A study can be awarded a maximum of 4 points for the Selection category, 2 points for the comparability category and 3 points for the Outcome/Exposure categories. Therefore, the maximum points a study can obtain is 9 which indicates a high-quality study.

Table 3. Characteristics of excluded studies

Study, year [Ref]	Type of study	Population	Metalloproteinase	Outcomes	Main findings	Reason for exclusion
**Masutomo, 2001 [26]**	Prospective observational NRCT	Male syrian hamsters	MMP-1, MMP-2, MMP-3, MMP-9, TIMP-1, TIMP-2	To analyze the effects of AT1 receptor blockade in response to losartan, on extracellular matrix degradation process in models of hypertrophic and dilated cardiomyopathy.	MMPs and TIMPs seem to be susceptible to responding to losartan.	Animal model
**Chiu, 2006 [27]**	Prospective observational NRCT	Cats	MMP-2, MMP-9	To investigate the effects of a higher dose of norepinephrine on cardiac remodeling.	MMP-9 activity was elevated, while MMP-2 activity was unchanged.	Animal model
**Kitz, 2019 [28]**	Prospective observational NRCT	Cats	MMP-2, MMP-3, MMP-13, TIMP-2	To analyze the pathogenesis of HOCM.	A negative correlation for MMP-2, MMP-13 and age in HOCM subjects.	Animal model
**Lim, 2021 [29]**	Prospective observational NRCT	Mice	MMP-2, MMP-3	To assess the atrial structural and electrophysiological alterations and blood biomarkers in HOCM subjects.	Increased MMPs levels in older mice.	Animal model
